# Perspective on fast-evolving photoacoustic tomography

**DOI:** 10.1117/1.JBO.26.6.060602

**Published:** 2021-06-30

**Authors:** Junjie Yao, Lihong V. Wang

**Affiliations:** a Duke University, Department of Biomedical Engineering, Durham, North Carolina, United States; b California Institute of Technology, Andrew and Peggy Cherng Department of Medical Engineering, Department of Electrical Engineering, Pasadena, California, United States

**Keywords:** photoacoustic tomography, optoacoustic imaging, volumetric imaging, high-speed imaging, optical ultrasound detection, wearable device, machine learning

## Abstract

**Significance:** Acoustically detecting the rich optical absorption contrast in biological tissues, photoacoustic tomography (PAT) seamlessly bridges the functional and molecular sensitivity of optical excitation with the deep penetration and high scalability of ultrasound detection. As a result of continuous technological innovations and commercial development, PAT has been playing an increasingly important role in life sciences and patient care, including functional brain imaging, smart drug delivery, early cancer diagnosis, and interventional therapy guidance.

**Aim:** Built on our 2016 tutorial article that focused on the principles and implementations of PAT, this perspective aims to provide an update on the exciting technical advances in PAT.

**Approach:** This perspective focuses on the recent PAT innovations in volumetric deep-tissue imaging, high-speed wide-field microscopic imaging, high-sensitivity optical ultrasound detection, and machine-learning enhanced image reconstruction and data processing. Representative applications are introduced to demonstrate these enabling technical breakthroughs in biomedical research.

**Conclusions:** We conclude the perspective by discussing the future development of PAT technologies.

## 1 Introduction

**Fig. 1:**
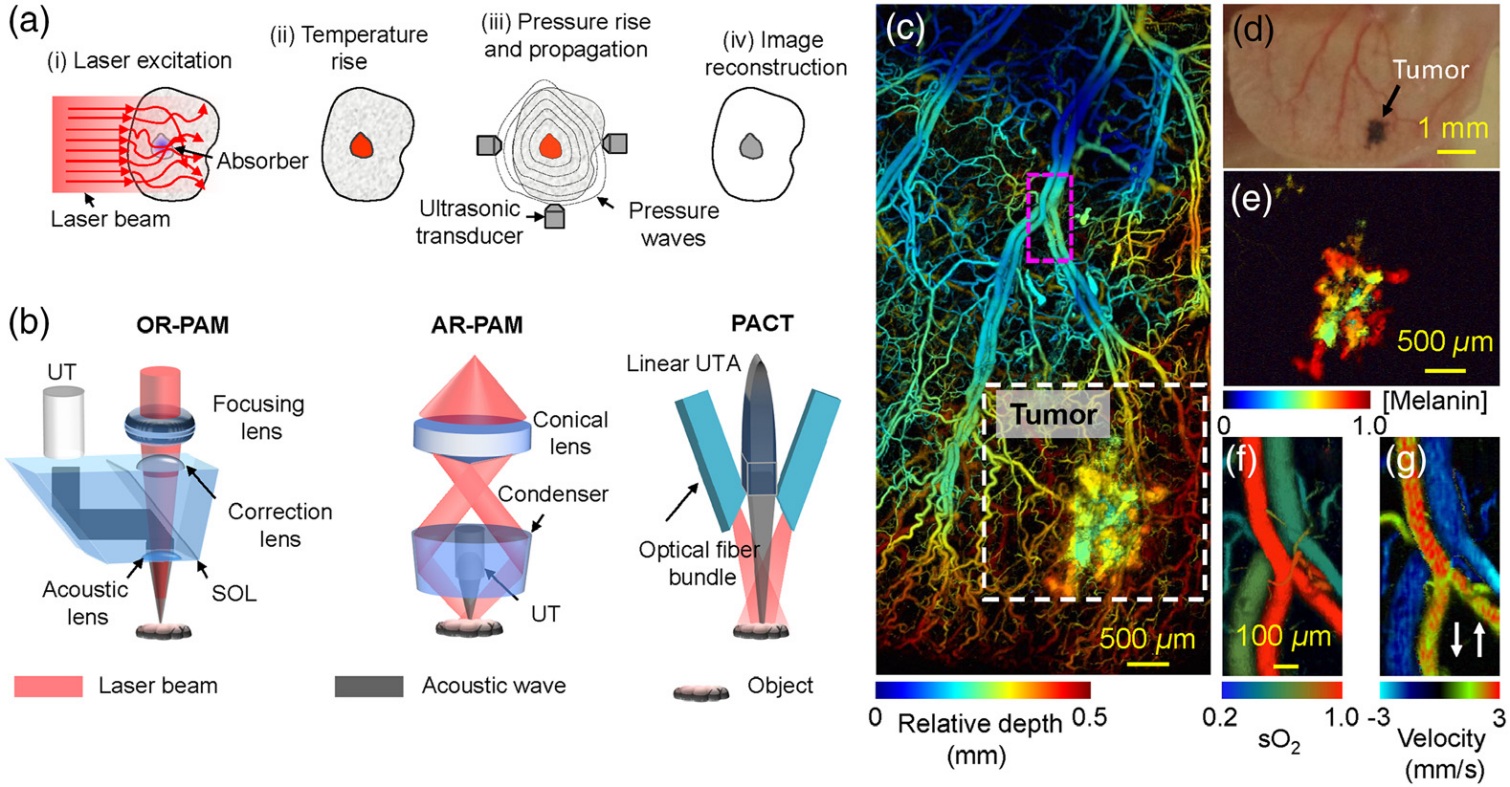
Principles, implementations, and representative applications of PAT. (a) Working principle of PAT, from the laser excitation to the image reconstruction. (b) Three representative implementations of PAT: optical-resolution photoacoustic microscopy (OR-PAM), acoustic-resolution photoacoustic microscopy (AR-PAM), and photoacoustic computed tomography (PACT) with a linear ultrasound transducer array. SOL, silicone oil layer; UT, ultrasound transducer; UTA, ultrasound transducer array. (c) OR-PAM image of the microvasculature of a mouse ear bearing a xenotransplanted B16 melanoma tumor (white dashed box) at 584 nm. Depth is coded by colors: blue (superficial) to red (deep). (d) White light photograph of the mouse ear. (e) OR-PAM image of the melanoma at 600 nm. Blood vessels are invisible due to the relatively weak absorption of hemoglobin at this wavelength. (e) OR-PAM image of oxygen saturation (${\mathrm{sO}}_{2}$) of the principal arterial-vein pair. (f) OR-PAM image of the blood flow velocity of the principal arterial-vein pair. The directions of positive and negative flow are defined by the arrows. Reproduced with permission from Ref. 2.

In the recent decade, photoacoustic tomography (PAT, also referred to as optoacoustic tomography or thermoacoustic tomography) has emerged as one of the fastest-growing imaging technologies and has become an enabling tool in many fundamental and translational studies, particularly for early cancer diagnosis, functional brain imaging, drug delivery monitoring, and interventional procedure guidance.^1^ The imaging process in PAT typically starts with a short laser pulse that illuminates biological tissue. As the excitation photons propagate through the tissue, some are absorbed by endogenous or exogenous biomolecules, and their energy is partially or completely converted into heat and thus a transient temperature rise, through nonradiative relaxation of excited molecules [Fig. 1(a)]. Generally, biomolecules with a lower or zero fluorescent quantum yield and a larger Grüneisen parameter have more efficient thermal conversion. When the excitation laser pulse width satisfies both thermal and stress confinement, the resultant initial pressure rise is proportional to the transient temperature rise via the thermoelastic effect.^3^ The pressure wave is then detected outside the tissue by an ultrasonic transducer or transducer array to form a tomographic image that maps the optical energy deposition inside the tissue. PAT has a 100% relative sensitivity to small optical absorption variations, which means a given percentage change in the optical absorption coefficient yields the same percentage change in the PA signal amplitude.^4^ Because PAT does not rely on fluorescence emission, which usually has a quantum yield much <100%, it can image nearly all molecules, fluorescent or not.^5^^–^^7^

Although PAT has been implemented in numerous configurations and tailored for diverse applications, its basic principles and major components remain similar. A typical PAT system includes (i) a short-pulsed laser or multiple lasers at one or more optical wavelengths for efficient PA wave generation, (ii) a wideband ultrasonic transducer or transducer array for PA signal detection, (iii) a data acquisition system for signal amplification, filtering, and digitization, (iv) an electronic system for trigger synchronization and data collection/streaming, and (v) a computational system for data processing, image reconstruction, and functional information quantification. So far, PAT has been implemented with two major image formation methods [Fig. 1(b)]. The first method, direct image formation (commonly referred to as photoacoustic microscopy, or PAM), is based on mechanical scanning of a focused excitation light beam and a focused single-element ultrasound transducer. A focused ultrasound transducer usually provides better detection sensitivity than a flat transducer. PAM can be further classified into optical-resolution PAM (OR-PAM) and acoustic-resolution PAM (AR-PAM), depending on the focal spot size of the optical excitation and acoustic detection.^8^ The second method, inverse reconstruction image formation (commonly referred to as photoacoustic computed tomography, or PACT), is based on wide-field light illumination and parallel acoustic detection by a multi-element ultrasound transducer array. Each transducer element can be approximated as a point detector with a large acceptance angle. Compared with PAM, PACT typically has a higher imaging speed and greater penetration, but lower spatial resolutions. Other PAT implementations, such as photoacoustic endoscopy, a miniaturized implementation of PAT for internal organ or intravascular imaging, can be implemented in either a PAM or PACT configuration.^9^^–^^15^ The imaging performance of major PAT implementations is summarized in our previous tutorial article.^3^ Readers are also referred to a practical guide for implementing PAT systems.^2^

Seamlessly integrating the optical excitation with acoustic detection, PAT has several advantages over other high-resolution optical imaging technologies: (i) PAT is maximally sensitive to the rich optical absorption contrast of biological tissue, and it is inherently well suited for anatomic, functional, and molecular imaging [Fig. 1(c)]; (ii) because biological tissue is more transparent to sound than to light in terms of the scattering mean free path, PAT provides far greater penetration depth than optical microscopy; (iii) because of the high scalability of optical excitation and ultrasound detection, PAT can be implemented in many different configurations, providing multi-scale observation of the same biological process with a consistent contrast mechanism; and (iv) PAT is functionally complementary to and engineeringly compatible with other imaging modalities, especially ultrasound imaging. PAT-capable multi-modal imaging can provide a more comprehensive understanding of biological phenomena.

PAT has gained tremendous momentum in the last decade, driven by innovations in high-power lasers, high-sensitivity ultrasound detection, high-speed scanning, large-scale computation, nanotechnology, protein engineering, and machine learning. In our tutorial published in 2016,^3^ we systematically introduced the foundation of PAT technologies, including the imaging principles from light to sound, the implementations at different length scales, and representative applications in life sciences. For readers interested in developing and/or applying PAT for biomedical research, our tutorial and other comprehensive review articles can provide a practical guide.^16^^–^^21^ Built upon our tutorial, this perspective aims to provide an update on the developments in PAT technologies in the last several years. Limited by the paper length, we are not able to cover all the exciting advances in PAT but will focus on several breakthroughs that have allowed new imaging capabilities not available to traditional PAT systems, including (i) volumetric PAT of deep tissues with nearly isotropic resolution, using a 2D ultrasound array; (ii) high-speed PAT with microscopic resolution, wide field-of-view (FOV), and functional imaging capability; (iii) high-sensitivity PAT with optical ultrasound detectors that have small sizes, wide bandwidth, and high transparency; and (iv) novel image reconstruction and data processing methods enabled by large-scale computation or machine learning, with improved image quality and quantitative accuracy. We introduce these PAT innovations in the context of the longstanding engineering challenges, summarize their much-improved imaging performance (usually by orders of magnitude over traditional PAT), and present the representative applications in fundamental research and translational studies. We conclude with a brief discussion of remaining challenges and future developments in PAT.

## 2 Technical Advances in PAT

The technological development in PAT has been fueled by advances in almost every key system component, from hardware to software, such as light sources with higher power, higher repetition rate, wider wavelength tuning range, and lower cost; novel ultrasound detectors with higher sensitivity, larger frequency bandwidth, and lower cost; and advanced image reconstruction algorithms with reduced artifacts, higher computation speed, and better quantification accuracy. For example, there has been a strong interest in using low-cost laser diodes and light-emitting diodes for PAM and PACT.^22^ The low-cost light sources typically have much lower pulse energy (less than a few mJ), longer pulse width (tens to hundreds of ns), and lower spatial/temporal coherence, compared with the Class IV pulsed lasers typically used in PAT, but they can substantially reduce the system cost, improve the portability, and thus facilitate the technical translation. The diode-enabled PAT systems have been used for a wide range of applications where the imaging depth or temporal resolution, spatial resolution, and/or the spectroscopic measurement accuracy can be relaxed, including needle biopsy guidance,^23^ melanoma imaging,^24^ skin implant monitoring,^25^ and human finger imaging.^24^

Limited by space, we will focus on several important developments that have overcome the longstanding limits in traditional PAT. Interested readers are referred to comprehensive review articles that provide in-depth analyses and discussions on low-cost light sources,^22^^,^^26^^–^^28^ novel ultrasound sensors,^21^^,^^29^^,^^30^ PA contrast agents,^29^^,^^31^^,^^32^ PA endoscopy,^33^^,^^34^ deep learning enhanced PAT,^35^^–^^37^ as well as clinical translation of PAT.^22^^,^^38^^–^^41^

### 2.1 Volumetric PACT with High-Speed and Isotropic Resolutions

PAT is inherently capable of volumetric or three-dimensional (3D) imaging, benefiting from the time-resolved detection of the acoustic waves that provide the depth information of the targets. For PAM, in which a single laser pulse generates a 1D depth-resolved image, 2D raster scanning is employed to obtain a 3D image; for PACT with a 1D transducer array, in which a single laser pulse generates a 2D cross-sectional image, orthogonal scanning along the elevational direction is needed to obtain a 3D image. We will discuss the new developments in PAM in a later section, but here we focus on volumetric PACT. While traditional volumetric PACT has been widely used for functional brain imaging, small-animal whole-body imaging, and breast cancer diagnosis in humans, the major drawbacks include the long imaging time needed for mechanical scanning and the anisotropic spatial resolution (much worse elevational resolution) determined by the cylindrical focusing of the transducer elements.

To accelerate the speed and improve resolution symmetry of volumetric PACT, recent efforts have concentrated on applying 2D ultrasound transducer arrays coupled with high-power laser sources and 3D image reconstruction. For PACT with a 2D transducer array, a single laser shot can theoretically generate a 3D image,^42^^,^^43^ and the resolutions can be nearly isotropic at the center of the FOV or the well-resolved FOV. In practice, however, 2D transducer arrays typically lack enough active elements to satisfy the spatial Nyquist sampling over a large volume, limited by the transducer fabrication complexity and the number of data acquisition channels. Thus, multiplexed data acquisition (electronic scanning)^44^ and rotational scanning^45^ are typically needed to improve the spatial sampling density. Moreover, because repeated wide-field illumination may cause tissue damage due to accumulated heating,^46^ the optical fluence ($J/m^{2}$) per pulse and the average fluence rate ($W/m^{2}$) on the tissue surface need to be carefully controlled.^46^

**Fig. 2:**
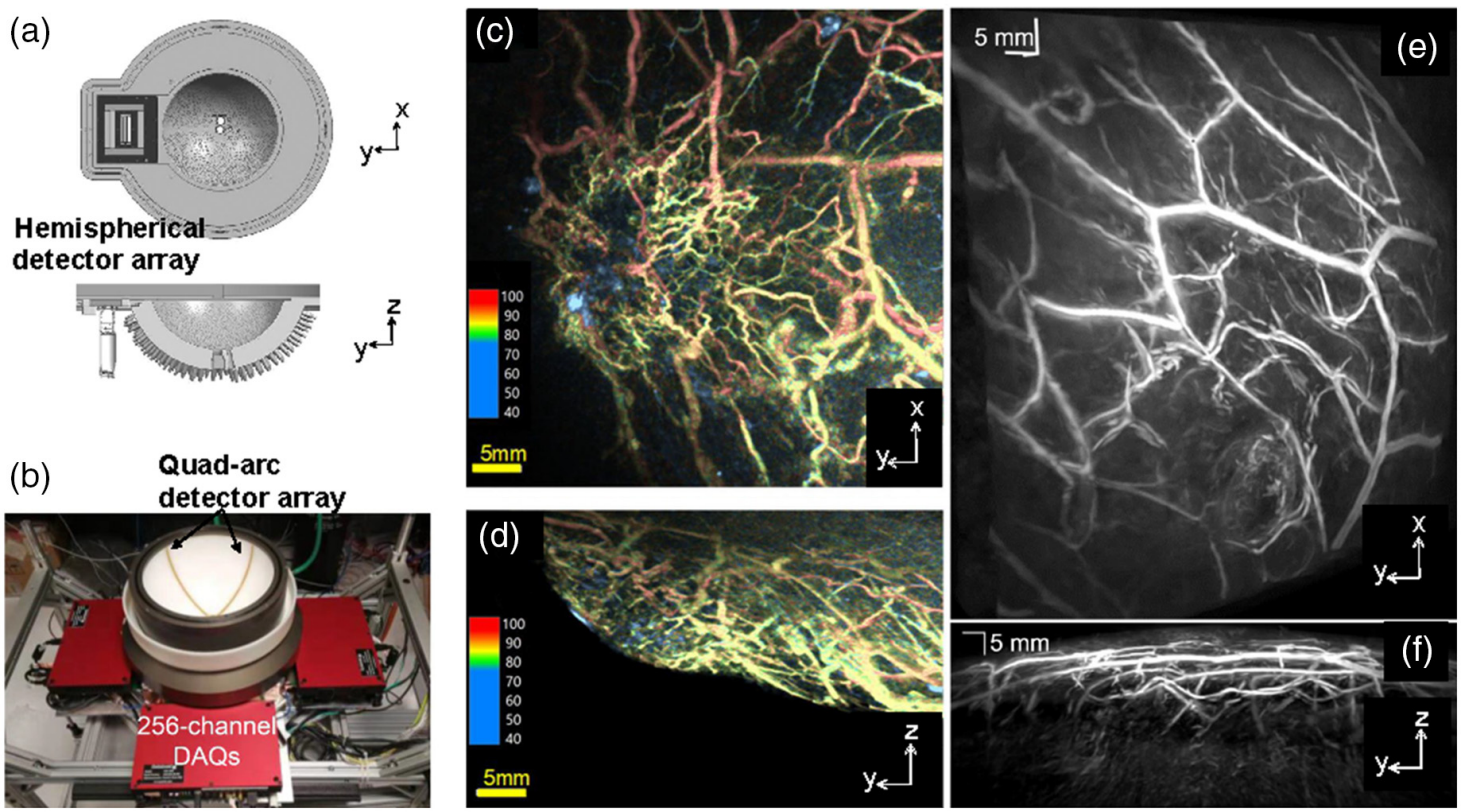
Volumetric PACT with 2D ultrasound array. (a) Schematic of the hemispherical array with 512 elements sparsely arranged over the sensor surface.^47^ (b) Schematic of the quad-arc array with 1024 elements densely arranged along four arcs (with a separation of 90 deg) that are mounted on a hemispherical surface.^49^ (c)-(d) Projection images of the human breast vascular oxygenation obtained by the volumetric PACT in (a), with a total scanning time of 120 s. (e)–(f) Projection images of the human breast vasculature obtained by the volumetric PACT in (b), with a total scanning time of 10 s. Adapted with permissions from Refs. 47 and 49.

Different types of 2D transducer arrays have been explored for volumetric PACT, mostly based on piezoelectric materials, as summarized in Table 1. To maximize the detection aperture, several groups have explored the feasibility of spherical ultrasound transducer arrays, usually with the transducer elements sparsely distributed over the array surface.^47^^,^^49^^,^^50^ Compared with the planar 2D array,^53^^,^^54^ the spherical array can provide higher spatial sampling density around its center volume and better visualization of 3D structures with different orientations. Matsumoto et al.^47^ developed a volumetric PACT system using a sparse hemispherical detector array that is scanned in a spiral pattern [Fig. 2(a)], which can provide detailed blood oxygenation images on the breast skin surface but suffers from slow imaging speed and low penetration depth [Figs. 2(c) and 2(d)]. Similarly, Schoustra et al.^50^ upgraded the Twente Photoacoustic Mammoscope using 12 arc-shaped transducer arrays arranged over a hemi-spherical surface, which can provide 3D vascular images of healthy breasts within four minutes. To accelerate the imaging speed, the Razansky group has developed a volumetric PACT system using a 2D ultrasonic transducer array with 256 elements densely arranged on a partial cup, which has achieved a 3D imaging rate of ∼50  Hz.^48^ This system has been implemented in both desktop and handheld configurations^55^^,^^56^ and has been applied to capture in real time the heart beating of a mouse and the neuronal activities of a swimming zebrafish and a GCaMP-expressing mouse brain.^42^^,^^55^^,^^57^ However, without performing additional scanning, such a transducer arrangement results in a small well-resolved FOV (∼4-mm diameter) and can only be used to study small animal organs, such as the heart and brain. Moreover, the limited view aperture (i.e., <2π steradian solid angle) can further compromise the image quality when a higher imaging speed is required.

**Table 1:** Comparison of representative 2D ultrasound arrays in volumetric PACT.

2D ultrasound array	Hemi-spherical	Cup	Quad-arc	12-arc	Fabry–Perot
Array radius (mm)	70	40	130	120	25 (max)
Number of elements	500	256	1024	384	$100/{\mathrm{mm}}^{2}$
Scanning scheme	Rotational	None for small FOV	Rotational	Rotational	Raster
		Spiral for large FOV			
Element shape	Circular	Square	Rectangular	Square	Circular
Element size (mm)	1.5	3	0.6 × 0.7	3.5 × 3.5	0.068
Element pitch (mm)	10	3.13	0.74	4.9	0.068
Central frequency (MHz)	4	4	2.25	1	11
Receiving bandwidth (%)	>100	100	>98	100	>100
Noise equivalent pressure (Pa)	0.5	1	5	Not available	200
References	47	48	49	50	51 and 52

To simultaneously improve the spatial sampling and imaging speed over a large FOV, the Wang group has reported a novel design with a quad-arc-shaped 2D transducer array, which has 1024 elements and one-to-one mapped signal amplification and data acquisition [Fig. 2(b)].^49^ By rotating the quad-arc-shaped array by 90 deg, the volumetric PACT system can provide a large well-resolved FOV (diameter>100  mm) and ∼2π steradian solid angle, with nearly isotropic resolution of 370 to 390  μm. It takes only 2 to 10 s to generate a volumetric image, depending on the targeted FOV, which is much faster than the previously reported systems. The newly developed volumetric PACT system has been applied for imaging a human breast within a single breath hold [Figs. 2(e) and 2(f)]. So far, this is the volumetric PACT system with the largest well-resolved FOV and the highest speed. Nevertheless, the imaging speed can be further improved by adopting pulsed lasers with a higher repetition rate (>10  Hz) as well as faster rotational scanning stages. Meanwhile, functional and molecular imaging capability remains to be demonstrated with high-speed, wavelength-tunable light sources. However, to comply with the laser safety standard,^46^ a higher laser repetition rate would lead to a lower maximum permissible exposure ($\mathrm{mJ}/{\mathrm{cm}}^{2}$) on the tissue surface, and thus a lower signal-to-noise ratio. In other words, a higher imaging speed in volumetric PACT may come at the cost of the final image quality and penetration depth.

The imaging characteristics of several representative volumetric PACT systems are summarized in Table 2.

**Table 2:** Comparison of representative volumetric PACT systems.

Volumetric PACT systems by institute	Kyoto University	University of Zurich	Caltech	University of Twente	UCL
Array shape	Hemi-spherical	Cup	Quad-arc	12-arc	Fabry–Perot
Lateral resolution (μm)	270	200	390	1060	100
Axial resolution (μm)	270	200	370	960	100
Diameter of well-resolved FOV without scanning (mm)	N/A	4	N/A	N/A	N/A
Diameter of well-resolved FOV with scanning (mm)	140	80	>100	>50	10
Laser repetition rate (Hz)	10	100	10	10	200
Imaging time (second)	120	45	2-10	240	10
Imaging depth (cm)	1	2	4	2.2	1
References	47	48	49	50	51 and 52

In addition to 2D ultrasound transducer arrays based on piezoelectric materials, optical ultrasound detectors, such as the Fabry–Perot interferometer,^58^^,^^59^ micro-ring resonator,^60^ and Bragg grating fiber,^61^^–^^63^ have been actively explored for volumetric PACT. Compared with piezoelectric transducers, optical ultrasound detectors often have smaller size (<100  μm), larger detection bandwidth and receiving angle, higher detection sensitivity per unit area, better transmittance of the PA excitation light, and simpler PA signal readout, all of which can help improve the resolution and penetration depth of volumetric PACT. We will discuss the developments in optical ultrasound detectors in a later section.

### 2.2 High-Speed Photoacoustic Microscopy over a Large FOV

Biological functions occur on a wide range of temporal and spatial scales, which requires imaging technologies to provide best-matched imaging speeds and FOVs. For example, a single neuron action potential lasts for 1 to 2 ms along a 10-μm-diamter axon, neurovascular coupling happens within hundreds of milliseconds over a functional circuit with a 100-μm radius, and the resting-state functional connectivity between the brain’s sub-regions occurs within tens of seconds over a millimeter-level radius. Configured to work in 1D, 2D, or 3D imaging modes, different implementations of PAT offer a wide range of imaging speeds with associated tradeoffs.^64^ In this section, we will focus on new developments in PAM that can offer high imaging speed, large FOV, and functional imaging capability.

For PAM, different scanning mechanisms can be employed according to the desired imaging speeds.^8^ Unlike confocal or two-photon microscopy, PAM does not require depth scanning for 3D imaging due to its time-resolved acoustic detection. When high-speed imaging is needed in OR-PAM, the focused excitation laser beam can be raster-scanned within the acoustic focal spot (∼50  μm in diameter), which largely confines the FOV to single vessels.^65^^,^^66^ Alternatively, cylindrically focused or unfocused acoustic detection can enlarge the FOV—up to ∼40  mm in diameter as demonstrated thus far—at the expense of detection sensitivity.^67^^,^^68^ In order to achieve a high detection sensitivity over a large FOV, it is critical to maintain the confocal alignment of the optical excitation and acoustic detection. Recently, 1D or 2D water-immersible resonant MEMS (microelectromechanical systems) scanning mirrors that confocally steer both the excitation laser beam and the emitted acoustic beam^69^ have achieved a 2D imaging rate of 500 Hz and a 3D imaging rate of ∼1  Hz, with a moderate FOV of $∼3\times 3\;{\mathrm{mm}}^{2}$ and uncompromised detection sensitivity.^70^^–^^72^ By using a pulse-width-based, single-wavelength method or a Raman-shifter-based, two-wavelength method, MEMS-scanning OR-PAM can monitor the change in blood oxygenation of mouse brain *in vivo*.^71^^,^^73^ However, it is challenging for the resonant MEMS scanning mirrors to provide a larger FOV without sacrificing the system’s detection sensitivity, and the scanning range drops sharply when the scanning frequency deviates from the resonant frequency.

**Fig. 3:**
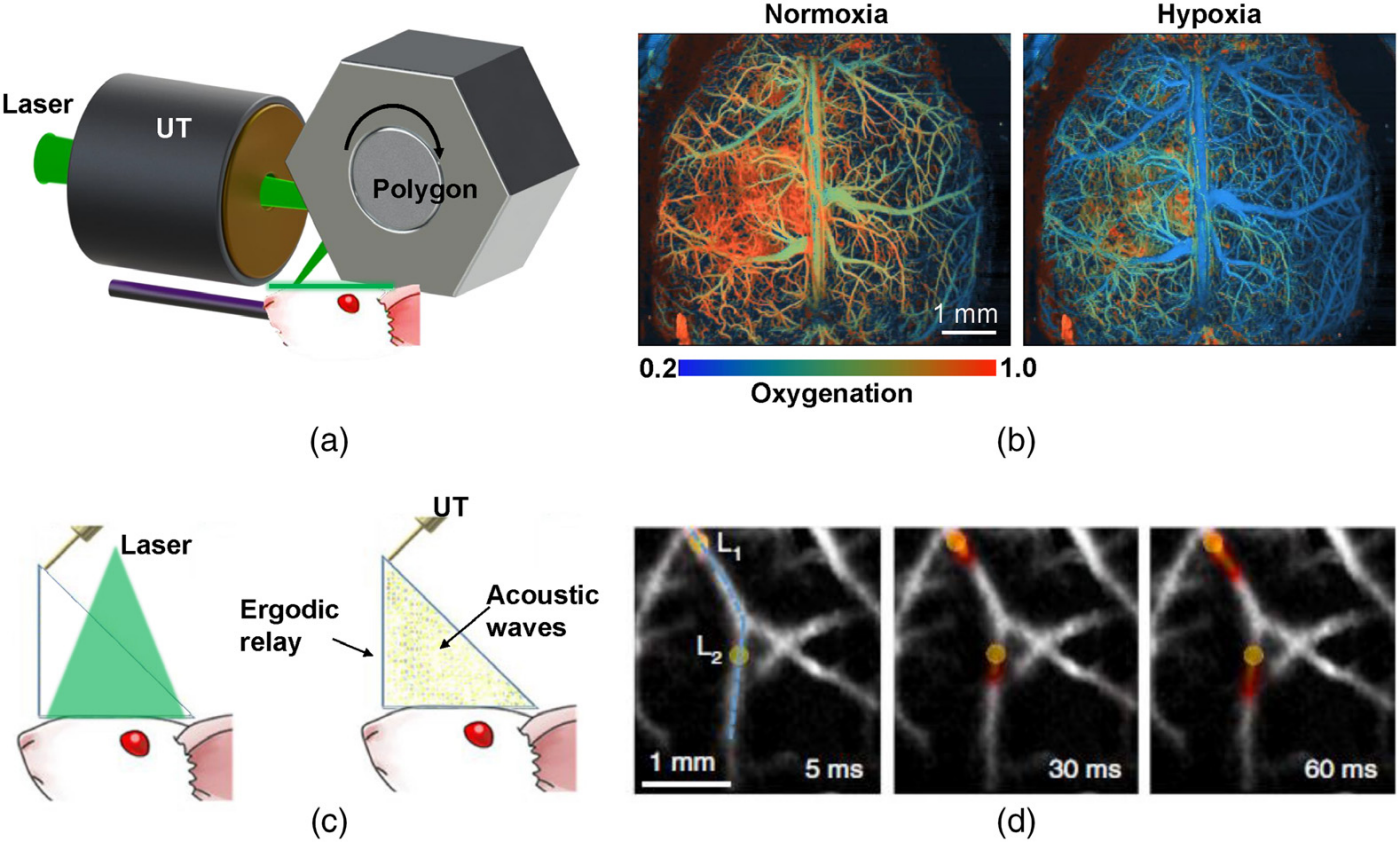
High-speed PAM with novel scanning and non-scanning approaches. (a) Schematic of the high-speed PAM using a water-immersible polygon scanner, in which a single rotation of the polygon scanner provides six repeated 2D scans.^74^ UT, ultrasound transducer. (b) High-speed imaging of the mouse brain under hypoxia challenge obtained by the system in (a), showing reduced blood oxygenation. (c) Schematic of the high-speed PAM using wide-field light illumination and a single-element ultrasound detector through an ergodic relay.^75^ (d) High-speed tracking of arterial pulse wave obtained by the system in (c), showing the heated blood (coded in color) flowing in the vessels. Adapted with permissions from Refs. 74 and 75.

To address the tradeoff between the scanning speed and scanning range of MEMS scanners, a recent work by Lan et al.^74^ has reported the use of a water-immersible polygon mirror scanner in OR-PAM that has achieved a 2D imaging rate of 1.2 kHz over a 12-mm scanning range and a 3D imaging rate of 1 Hz over a $12\times 12\;{\mathrm{mm}}^{2}$ FOV [Fig. 3(a)]. The polygon scanner with six facets is driven by a rotational DC motor, with each rotation providing six repeated 2D scans. Unlike the resonant MEMS mirror, the polygon scanner can maintain its large scanning range at different scanning frequencies, which is critical for imaging large organs, such as the blood oxygenation change of the whole mouse cortex [Fig. 3(b)]. By combining the polygon scanner with a Raman-shifter-based, two-wavelength laser, Chen et al.^76^ have demonstrated high-speed functional imaging of the hemodynamic response of the entire mouse ear to epinephrine, a commonly used vasoconstrictor. Nevertheless, one drawback of the polygon scanner is the lack of adjustment of its scanning range. Once the optical path is constructed, the scanning range is determined and difficult to change, which poses a waste of scanning time on small targets. Different scanning mechanisms used in high-speed OR-PAM are compared in Table 3.

**Table 3:** Comparison of scanning mechanisms in OR-PAM.

Scanning methods	B-scan rate (Hz)	Scanning range (mm)	Detection Sensitivity^a^	Transducer focusing	Ref
Mechanical motor	1	10	+++	Spherical	77
Voice-coil scanner	40	5	+++	Spherical	78
Galvo scanner (unfocused transducer)	100	∼6	+	Unfocused	79
Galvo scanner (2D focused transducer)	180	<0.1	+++	Spherical	65
Galvo scanner (1D focused transducer)	50	20	+	Cylindrical	68
Water-immersible MEMS scanner	400	3	++	Spherical	80
Water-immersible polygon scanner	1200	12	++	Spherical	74

a More plus signs indicate better detection sensitivity.

For AR-PAM, the imaging speed is mainly limited by the mechanical scanning speed and the pulse repetition rate of the high-pulse-energy laser, the latter of which is limited by laser safety on the tissue. In AR-PAM, mechanical scanning by a step motor or a voice-coil scanner can be used, with a scanning step size ∼10 times that used in OR-PAM.^8^ A 2D imaging rate of 40 Hz has been achieved by AR-PAM over a scanning range of ∼9  mm, sufficient to capture the oxygenation dynamics in a mouse heart within a heartbeat.^81^ Recently, the 2D water-immersible MEMS scanning mirrors have also been adapted to improve the imaging speed of AR-PAM by ∼10-fold, with an FOV of $2\times 2.5\;{\mathrm{mm}}^{2}$.^82^^–^^84^ When integrated with additional mechanical scanning to “stitch” the MEMS scanning area, AR-PAM can image a $30\times 30\;{\mathrm{mm}}^{2}$ area within 70 s.

While the above fast-scanning–based approaches have significantly improved the imaging speed of PAM systems, they are fundamentally limited by the laser’s pulse repetition rate when the spatial Nyquist sampling needs to be satisfied. This limitation is particularly true for high-speed OR-PAM, which often requires a small scanning step size of <2  μm. For example, for the recently published polygon-scanner–based PAM system,^74^ the laser’s maximum pulse repetition rate is 800 kHz and the B-scan (i.e., the fast-scanning axis) rate can reach as high as 2000 Hz over a 10-mm scanning range. However, to satisfy the Nyquist sampling theorem, the B-scan rate is limited to only 200 Hz if the FOV is kept the same, much lower than the maximal achievable speed. One way to increase the scanning speed over a large FOV is to increase the scanning step size at the cost of effective spatial resolution. Sparse sampling has thus become a necessary compromise when imaging speed needs to be increased.^85^

To relax the requirement on the laser’s pulse repetition rate, one solution is non-scanning PA imaging based on an ergodic relay, which can simultaneously encode all of the PA signals from a large FOV according to their unique time-delay characteristics.^75^^,^^86^^–^^88^ In a recent work, Li et al.^75^ demonstrated a high-speed implementation referred to as photoacoustic topography through an ergodic relay (PATER). In PATER, for each single excitation laser pulse, the encoded PA signals can be detected in parallel via a single-element ultrasound transducer and then decoded mathematically to reconstruct a 2D projection image [Fig. 3(c)].^75^ With a point-by-point scanning calibration step, PATER has demonstrated a topographic frame rate of 2 kHz over a field of view of $6\times 7.5\;{\mathrm{mm}}^{2}$, and has been applied to image the blood pulse wave velocity and track the circulation of melanoma cells in the mouse brain [Fig. 3(d)]. Because no optical or acoustic beam scanning is needed in PATER, the imaging speed is essentially limited by the acoustic transit time within the ergodic relay. Nevertheless, the current calibration method lacks the depth information and thus only topographic images can be provided.

Limited by slow imaging speed and bulky system size, desktop PAM is mostly applied on small animals under anesthesia or human subjects with the targeted region fixed (e.g., arm, hand, or finger) to minimize the motion artifacts. Enabled by the elevated imaging speed and the resultant high imaging throughput, it has become possible to implement miniaturized PAM to image otherwise challenging targets prone to motion artifacts, such as brain functions of freely moving animals, longitudinal monitoring of rare circulating tumor cells of melanoma patients, and skin cancer screening of difficult regions such as the neck and back. In recent years, various PAM systems have been developed for handheld,^89^^–^^91^ wearable,^92^^–^^94^ and even head-mounted applications,^95^^,^^96^ thanks to the advances in high-speed scanning methods. All these technical innovations have allowed the miniaturization of PAM systems without sacrificing the imaging performance. For example, to capture normal brain functions, it is critically important to record the neural activities in freely behaving animals with high resolution and high throughput. Chen et al.^95^ have reported a wearable PAM system that is small enough to be mounted on the head of a freely moving rat. A miniaturized MEMS scanning mirror provided high-speed, high-resolution imaging of the brain’s hemodynamic activities during and post ischemia challenge. Remarkably, the motion artifacts were negligible during the 90-min imaging time.

### 2.3 Optical Detection of the Ultrasound Pressure

Piezoelectric ultrasound transducers still largely dominate the PAT technologies due to their wide availability, high detection sensitivity, low fabrication cost, and ease of use. However, optical ultrasound sensors have their unique advantages for PAT and have gained more momentum in recent years.^21^ Unlike ultrasonography, PAT does not need ultrasound transmission, and the PA signals are usually broadband, so optical sensors can be used in receiving-only mode, taking full advantage of their small size, large receiving angle, wide detection bandwidth, strong responsivity in the low frequency band, and good compatibility with PA light path. More importantly, the detection sensitivity of optical sensors usually has less dependence on the sensor size, which leads to better sensitivity than piezoelectric transducers of the same size, especially at higher frequencies (>2.5  MHz).^97^ Practically speaking, the optimal size of piezoelectric transducers used in PACT is equivalent to a half-wavelength on the FOV boundary. Further size reduction provides no clear benefit in spatial sampling density or receiving angular directivity.^10^ Therefore, when comparing the performance of optical ultrasound sensors with piezoelectric transducers, we suggest half-wavelength sized piezoelectric transducers shall provide a fair comparison unless the application is inherently space-constrained. For example, the optical sensors’ high detection sensitivity with a small form factor is particularly attractive for endoscopic and wearable PAT implementations, in which the working space is extremely limited. A thorough comparison of optical sensors and piezoelectric transducers can be found in the review article by Wissmeyer et al.^21^

So far, there have been two types of optical sensors demonstrated in PAT technologies: interferometric sensors and refractometric sensors. Taking advantage of the optical and acoustic interactions in the PA effect, these optical sensors often probe a single step in the PA signal generation and propagation process. The interferometric sensors typically have better detection sensitivity than the refractometric sensors.^21^ The above-mentioned Fabry–Perot interferometer, micro-ring resonator, and Bragg grating fiber are all interferometric sensors that target the last step in the PA signal propagation and have been applied in volumetric PACT and/or PAM as point-like detectors. Refractometric sensors often exploit the earlier steps in PA signal generation, such as the photothermal or thermoelastic effect in the tissues or coupling medium, and detect the change in probing light beam’s transmission, reflection, or deflection.^98^^–^^100^ Such changes, however, are usually small. While interested readers are referred to the comprehensive review article on the optical sensors in PAT technologies,^21^ we would like to discuss three important limitations of optical sensors—speed, scalability, and stability—as well as highlight some new studies aiming to address these limitations.

**Fig. 4:**
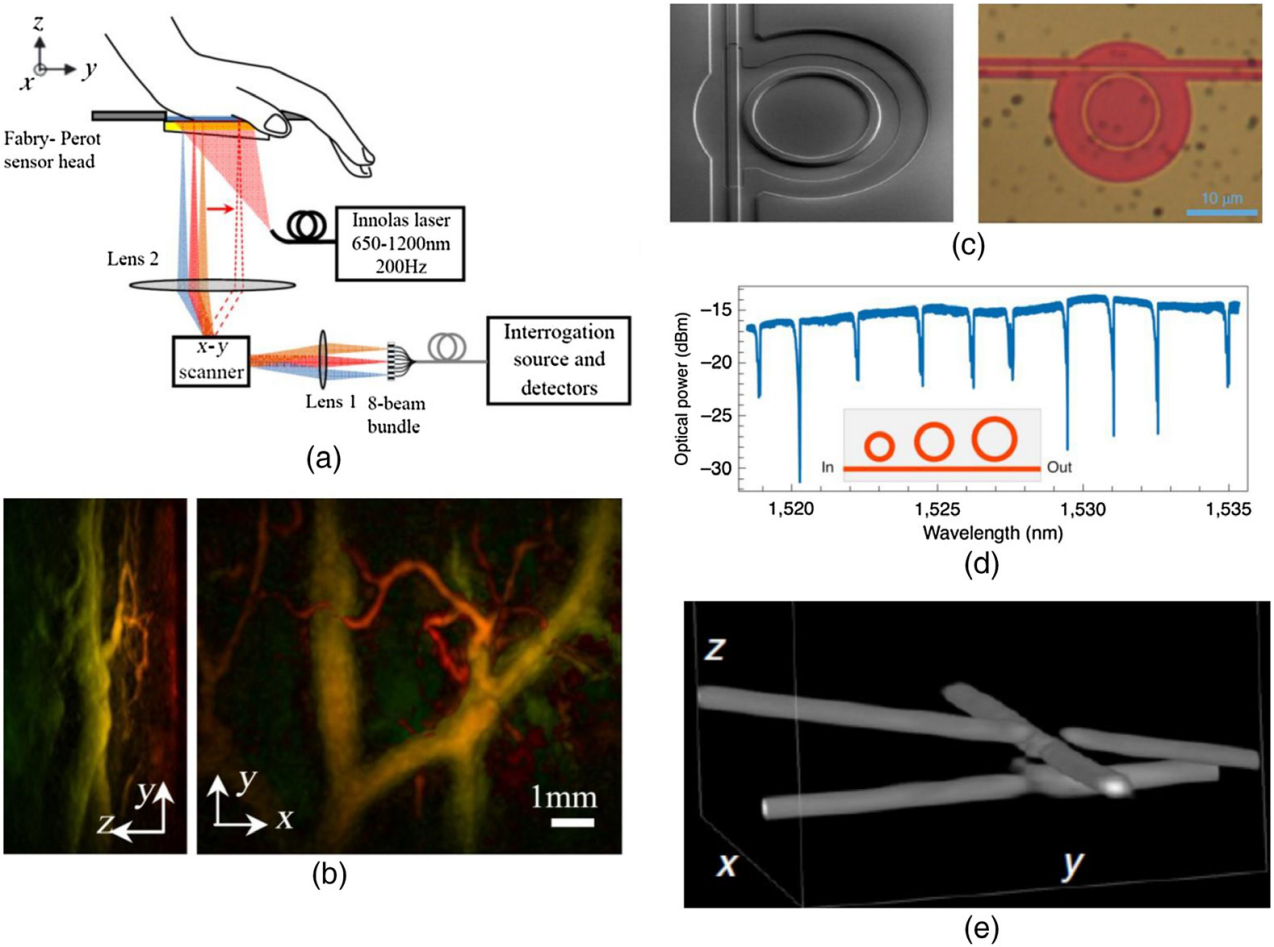
PAT with optical ultrasound sensors. (a) Schematic of a PACT system using a Fabry–Perot interferometer with eight parallel probing beams.^102^ (b) A 3D human palm image obtained the system in (a) with a total imaging time of 10 s. (c) Schematic and photograph of a micro-ring-resonator using silicon photonic technology.^60^ (d) The optical transmission spectrum of a multiplexed micro-ring-resonator array with 10 sensors. (e) Representative 3D image of three stacked polyamide sutures obtained by the micro-ring resonator in (a). Adapted with permissions from Refs. 60 and 102.

For the PACT systems based on a planar Fabry–Perot interferometer, the nearly isotropic spatial resolutions, approximately defined by the optical probing beam size, can be well maintained with 2D dense spatial sampling over the entire FOV.^51^ However, the imaging speed is traditionally limited by the point-by-point raster scanning of the probing beam and the low pulse repetition rate of the PA excitation laser at 50 Hz.^51^^,^^101^ A more recent work by the UCL group has demonstrated a 32-fold higher imaging speed by employing a total of eight parallel probing beams scanning simultaneously over the sensor [Figs. 4(a) and 4(b)].^102^ A customized, high-speed laser (200 Hz) also helps to improve the imaging speed. Such a speed-up strategy, however, has drastically increased the system’s complexity and cost, and the high-speed laser has relatively low pulse energy. Wide-field detection of the interference pattern on the sensor surface, using time-gated light illumination and a high-speed CCD camera, can potentially speed up the imaging as well.^103^^,^^104^

Other optical ultrasound sensors, including polymer micro-ring^60^ and Bragg-grating fiber,^61^ have recently been demonstrated as point-like detectors in PAT, often with sensor sizes that are orders of magnitude smaller than their piezoelectric counterparts. However, one major obstacle met by these optical sensors is the extreme difficulty in scaling up the production while maintaining consistent optical properties, such as the optical resonant wavelength, *Q* factor, and transmission efficiency. Unlike piezoelectric materials that allow the manufacturing of high-density arrays, it is difficult for optical sensors to be multiplexed. Slight fabrication inaccuracy, such as of the Fabry-Perot polymer’s thickness or the micro-ring’s diameter, would drastically change its operating parameters. This is particularly problematic for volumetric PACT, which requires parallel signal detection to improve the imaging speed. To address this issue, Westerveld et al.^60^ have developed a new micro-ring-resonator using silicon photonic technology [Fig. 4(c)]. As a proof of concept, a total of ten resonators can be fabricated onto a single optical bus waveguide [Fig. 4(d)]. This CMOS-compatible fabrication process may provide a viable path for scaling the optical sensor to a 2D array for high-speed volumetric PACT [Fig. 4(c)].

Another significant drawback of optical ultrasound sensors, particularly the interferometric sensors, is low stability in the biological environment. For example, the micro-ring resonator is sensitive to contamination on the sensor surface (e.g., dust, body fluid, or blood stain), which induces scattering and absorption loss, and the Fabry–Perot interferometer is sensitive to the environmental temperature drift, which changes the thickness and refractive index of the polymer spacer. Such instability in the biological environment often leads to fast degradation of the sensor sensitivity and prevents the use in longitudinal *in vivo* studies. To address this issue, Li et al.^105^ have developed a micro-ring resonator by soft nanoimprinting lithography, which has significantly improved stability for *in vivo* applications. The micro-ring resonator is encapsulated by a protection layer made of both optically and acoustically transparent polydimethylsiloxane (thickness 5  μm). By isolating the micro-ring and waveguide from the potential contaminants (e.g., blood), the micro-ring resonator has demonstrated impressively stable performance when implanted on a mouse cortical surface for 28 days. Similarly, Westerveld et al.^60^ recently demonstrated a micro-ring resonator using a thin layer of acoustic membrane to isolate the ring structure from the environment, which can potentially improve the sensor’s stability in water. To overcome the thermal stability of the Fabry–Perot interferometer, Chen et al.^106^ have incorporated an additional heating light source at 650 nm into the interferometer, which can modulate the polymer spacer’s thickness and thus compensate for the temperature-induced resonant spectral shift. Such thermal compensation can be performed in real time by a closed-loop feedback.

### 2.4 Deep Learning Enhanced Image Reconstruction and Processing

Like many other technologies, PAT’s developments have been incorporating the fast-evolving deep learning enabled by the prevalence of graphical processing unit (GPU) capabilities.^107^^–^^116^ Deep learning is well-suited for addressing some long-standing challenges of PAT, such as improving ill-posed reconstruction, removing limited-view artifacts, denoising channel data, improving diffraction-limited spatial resolution, and upsampling sparse input data. Many of these efforts have proven to be promising when traditional solutions either fail or make only incremental progress. There have been several excellent reviews on the history and status of deep learning technologies in PAT,^35^^,^^36^^,^^117^ to which we refer interested readers. A detailed comparison of different deep learning approaches in PAT can be found in the review article by Gröhl et al.^36^ Here we will highlight several of the most exciting advances.

**Fig. 5:**
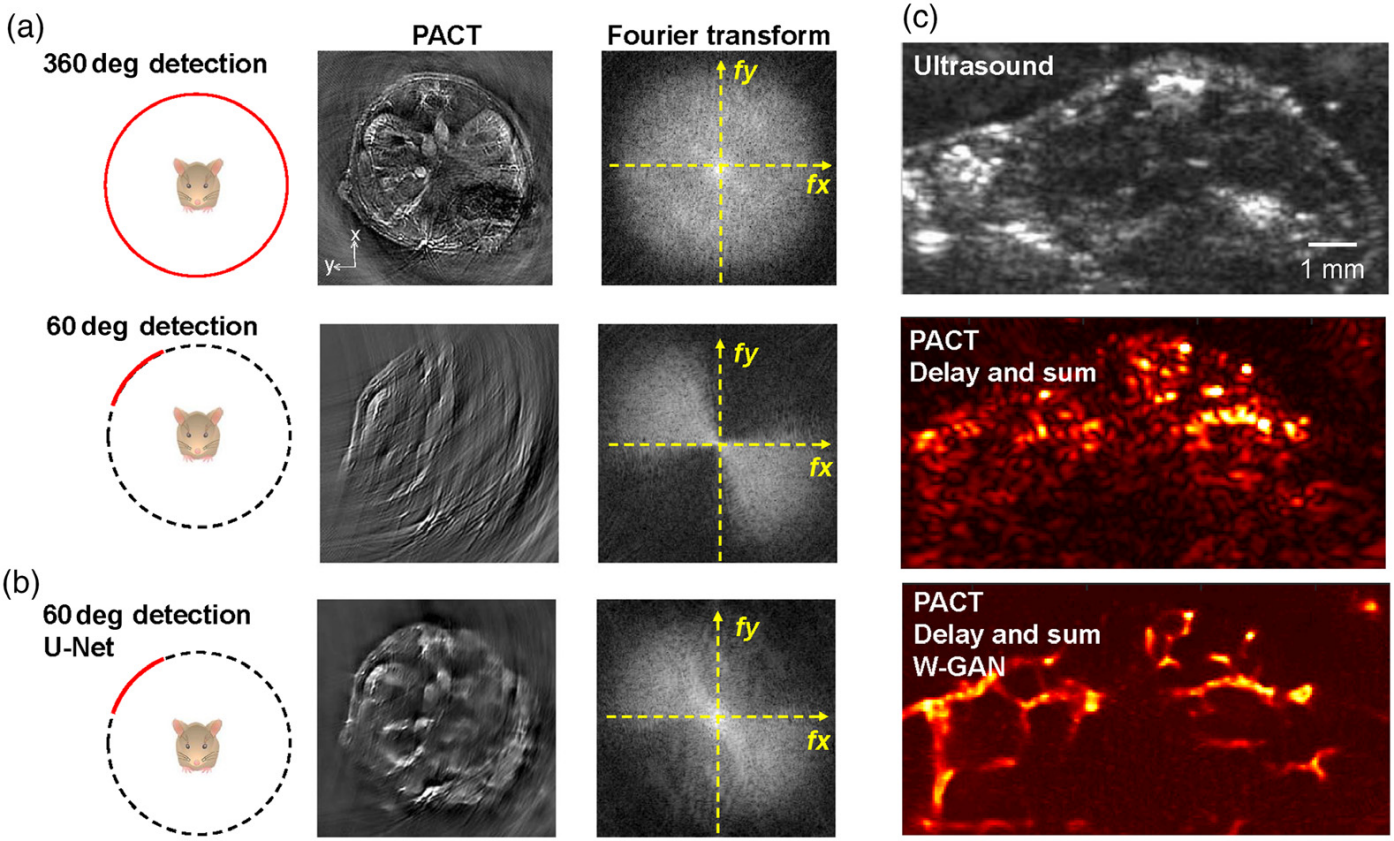
Limited-view PACT improved by deep learning methods. (a) Whole-body PACT images of a mouse and the corresponding spatial frequency spectra obtained by a ring-array system with a 360 deg or 60 deg detection angle range. The images were reconstructed using the traditional back projection method.^120^ (b) A U-Net–based deep learning method was used to reconstruct the PACT image with a 60 deg detection angle. (c) A comparison of PACT images obtained by a linear array before and after a GAN method was used to reduce the limited-view artifacts.^121^ Adapted with permissions from Refs. 120 and 121.

There is a clear difference between the deep learning formulation in PACT and PAM. In PACT, many challenges arise from solving the inverse problem, mostly with partial and/or sparse detection geometries.^118^ Deep learning in PACT can be used as (i) a pre-processing or post-processing step in the image reconstruction, (ii) replacement of the traditional image reconstruction altogether, or (iii) one integrated step in the iterative reconstruction. For example, Gutta et al. used a fully connected deep neural network (FC-DNN) as a pre-processing step to correct the sonograms acquired by each transducer channel and broaden the bandwidth of the received channel data.^119^ Davoudi et al.^120^ used a fully convolutional neural network (U-Net) to reconstruct the PACT data obtained by a ring array with limited view or sparse sampling, which resulted in improvements in both spatial frequency coverage and the final image quality [Figs. 5(a) and 5(b)]. For PACT with a linear transducer array, a stabilized generative adversarial network (GAN) model with gradient clipping has been employed as a post-processing step, which can reduce the limited-view and limited-bandwidth reconstruction artifacts of *in vivo* data [Fig. 5(c)].^121^ Another key area of research is integrating the deep learning into the PA forward operator for iterative–based image reconstruction, as demonstrated by Hauptmann et al.^122^^,^^123^ and Bioink et al.^124^ However, these iterative methods can be time–consuming.

Unlike PACT, PAM does not require inverse reconstruction, so deep learning models can directly map time-resolved input signals to output images, and improve imaging speed, signal-to-noise ratio, and spatial resolution. One of the major utilizations of deep learning in PAM is to improve sparsely sampled images, thereby shortening image acquisition time without substantially degrading image quality. For example, DiSpirito et al.^125^ have developed a modified fully dense U-Net architecture (FD U-Net), and demonstrated the feasibility to recover microvessels in the mouse brain by acquiring only 2% of the pixels required by the Nyquist sampling. For situations that lack ground truth data for model training, Vu et al.^126^ have proposed an innovative method that iteratively refines undersampled PAM images using a deep learning prior. This work is of particular interest because it does not require training on a large PAM dataset with ground truth. Deep learning has also recently been used by Song et al.^127^ to improve PAM images with extremely low excitation laser energy. Most recently, Sharma and Pramanikhave^128^ developed an FD U-Net to enhance the lateral resolution of AR-PAM, especially in the out-of-focus regions.

Notably, deep learning methods have also been investigated for improving quantitative PAT, which has been difficult for deep-seated targets due to spectral coloring. Deep learning approaches have been developed to either better estimate the optical fluence at different wavelengths or completely replace the traditional spectral unmixing algorithms. For example, a sequential-learning recurrent neural network has been used to predict eigen-fluence maps in deep tissue,^129^ which were subsequently used for linear unmixing of the oxy- and deoxy-hemoglobin concentrations.^129^ In another work, Gröhl et al.^130^ applied a fully connected neural network on multi-spectral PA images, which improved the quantification accuracy of blood oxygenation estimations on phantoms and *in vivo* porcine brain. Further, Bench et al.^131^ applied a 3D encoder-decoder style neural network to predict volumetric blood oxygenation; however, this methodology has not yet been adapted to *in vivo* data due to the complexity of tissue’s optical properties.

Nevertheless, one obstacle to the broad adoption of deep learning in PAT is the heavy reliance on simulation data and the lack of large, open-source repositories of *in vivo* data. The gap between simulation data and *in vivo* data makes model extrapolation to *in vivo* applications difficult. Potential solutions to address this obstacle are for the community to (i) create a large, open-source repository of various *in vivo* training examples or (ii) improve the quality of simulation data to better mimic *in vivo* cases. Ultimately, the incorporation of deep learning into PAT requires the training of robust models that can readily adapt to a variety of *in vivo* conditions—many of which, such as sparsely-sampled, limited-view, and limited-bandwidth detection, are in non-ideal environments.

## 3 Conclusion and Outlook

Harnessing the relevant advances in physics, chemistry, mathematics, and computer science, PAT has experienced its fastest development in the last decade and become the enabling technology in many biomedical studies. Previously, the technical innovations in PAT were often limited by the performance of key system components, such as the laser’s pulse repetition rate and the ultrasound transducer’s sensitivity. Although many engineering solutions were explored to address these long-standing technical challenges, they often require trade-offs between imaging parameters, such as the imaging speed versus the field of view, the spatial resolution versus the penetration depth, and the detection sensitivity versus the detector size. Thanks to advances in key technologies, such as high-power laser sources, fast scanning mechanisms, and miniaturized optical ultrasound sensors, the traditional tradeoffs in PAT technical development have become less constraining. The current perspective builds upon our previous tutorial on the fundamentals of PAT and highlights several key technical developments that have generated the most impact.

Innovations in volumetric PACT, with high speed and isotropic resolution, have addressed one of its most prominent technical hurdles precluding clinical potential. Full spatial sampling over a large FOV has enabled image quality similar to that of MRI or x-ray CT, particularly for breast cancer imaging. The functional and molecular imaging provided by PACT will likely complement the existing clinical imaging technologies and improve the detection specificity of malignant cancers. The technical advances in PAM have lifted the traditional tradeoffs between imaging speed, FOV, and detection sensitivity. Powered by super-fast pulsed lasers and novel scanning mechanisms, PAM has achieved high-speed, high-resolution imaging over the similar FOV of conventional CCD-based optical microscopes and can monitor the neurovascular coupling of the entire mouse cortex. The substantial improvement in imaging throughput has enabled implementations of PAM in portable and wearable formats, allowing for longitudinal monitoring of biological functions in freely moving animals or awake patients, with negligible motion artifacts. Both PACT and PAM can greatly benefit from the new advances in optical ultrasound sensors with small size, large detection bandwidth, and wide receiving angle. Innovations in the fabrication process, materials, and stabilization methods are critically important to address the limitations in the optical sensor’s speed, scalability, and stability. The relatively high detection sensitivity of small optical sensors is particularly beneficial for endoscopic and wearable PA applications. Moreover, the fast-evolving deep learning technologies have been quickly adopted in PAT to improve the signal-to-noise ratio, inverse image reconstruction, and image post-processing. For technical challenges in PAT difficult to address using hardware solutions, deep learning approaches may provide effective data-driven solutions that impose minimal impact on the system’s complexity and cost.

Looking forward, we expect that PAT will grow at an accelerating speed in both technology development and biomedical applications. With more commercially available PAT systems tailored for clinical practice, the user base will also experience a fast expansion in the next several years, resulting in a large number of published clinical studies. Of particular importance is the first FDA-approved PAT system by Seno Medical Instruments, Inc., which has paved the way for more commercial PAT systems to receive regulatory clearance. Developing low-cost PAT systems is an important step that can help improve its accessibility by the biomedical community.^22^ In particular, low-cost light sources such as laser diodes and light emitting diodes can significantly reduce the system cost of both PAM and PACT, and accelerate the technical translation to clinical practice. The success of commercial PAT products will in turn provide strong incentives for key industrial partners to develop products that are specially optimized for PAT, such as high-power, high-speed lasers with relaxed coherence; low-cost CMUT or PMUT arrays with a large number of elements;^30^ high-channel-number, high-speed data acquisition systems with built-in amplification capability; and high-speed GPU systems with large on-chip memory. Enabled by these updated system components, which are often the bottlenecks in PAT technology, the next wave of technological breakthroughs will naturally follow, including (i) real-time volumetric PACT systems for human imaging, such as breast cancer screening; (ii) high-speed, high-resolution PAM of neuronal activities enabled by novel voltage- or calcium-sensitive PA probes; (iii) highly-compact endoscopic and intravascular PAT enabled by optical ultrasound sensors; (iv) single-organelle or single-molecule PA imaging enabled by super-resolution mechanisms; (v) large-scale, high-speed 3D modeling of PA signal generation and propagation in a complex system; and (vi) robust quantitative PAT of tissue functions and molecular compositions enabled by deep learning approaches. Finally, we envision that, in the big data era, the next generation of PAT technologies will likely have artificial intelligence incorporated at every step of system development. The light illumination, ultrasound detection, scanning mechanism, data acquisition, and image formation can be optimized by the accompanying machine learning models, which will make it possible to achieve the next generation of smart PA technologies.
